# Atypical Hemolytic Uremic Syndrome following Acute Type A Aortic Dissection

**DOI:** 10.1155/2020/2467953

**Published:** 2020-03-03

**Authors:** Eigo Ikushima, Manabu Hisahara, Takuya Nishijima, Hikaru Uchiyama, Tatsushi Onzuka, Yoshie Ochiai, Tsuyoshi Muta, Shigehiko Tokunaga

**Affiliations:** ^1^Department of Cardiovascular Surgery, Japan Community Healthcare Organization (JCHO) Kyushu Hospital, 1-8-1, Kishinoura, Yahatanishi-ku, Kitakyushu, Fukuoka 806-8501, Japan; ^2^Department of Hematology/Oncology, Japan Community Healthcare Organization (JCHO) Kyushu Hospital, 1-8-1, Kishinoura, Yahatanishi-ku, Kitakyushu, Fukuoka 806-8501, Japan

## Abstract

Atypical hemolytic uremic syndrome (aHUS) is a thrombotic microangiopathy (TMA)-related disease that manifests as a triad of microangiopathic hemolytic anemia, thrombocytopenia, and acute kidney injury (AKI) and is caused by uncontrolled activation of the complement system. We report the case of a 61-year-old woman with acute type A aortic dissection that subsequently developed into aHUS. The hematologic disorders underlying aHUS improved after treatment with the complement inhibitor eculizumab. It is important to consider aHUS when a patient clinically develops a triad of microangiopathic hemolytic anemia, thrombocytopenia, and an increasing creatinine level following cardiovascular surgery.

## 1. Introduction

Atypical hemolytic uremic syndrome (aHUS) is related to thrombotic microangiopathy (TMA) and is diagnosed by clinical presentation of a triad of microangiopathic hemolytic anemia, thrombocytopenia, and acute kidney injury (AKI) with negative results for Shiga-toxin-producing *Escherichia coli* (STEC), thrombotic thrombocytopenic purpura (TTP), and other TMA-related diseases, such as autoimmune diseases [1].

Aortic dissection develops due to tearing of the intima of the aorta and bleeding within the aortic wall, resulting in its dissection. The risk factors for aortic dissection are hypertension, dyslipidemia, or connective tissue disorder, such as Marfan syndrome. According to the Stanford classification, it is classified into type A and type B based on whether the ascending aorta is involved. Acute type A aortic dissection (ATAAD) is life-threatening and needs emergency open surgical repair. Despite emergency surgery, early mortality is approximately 20% [2]. Because ATAAD itself and surgical treatment, including cardiopulmonary bypass, induced hypothermia, or the lower body circulatory arrest technique, are highly invasive, bleeding could provoke anemia and thrombocytopenia, while malperfusion or surgical stress could provoke AKI. However, aHUS following ATAAD has not been reported. We report the first case of a 61-year-old woman with ATTAD who subsequently developed aHUS.

## 2. Case Presentation

**Figure 1:**
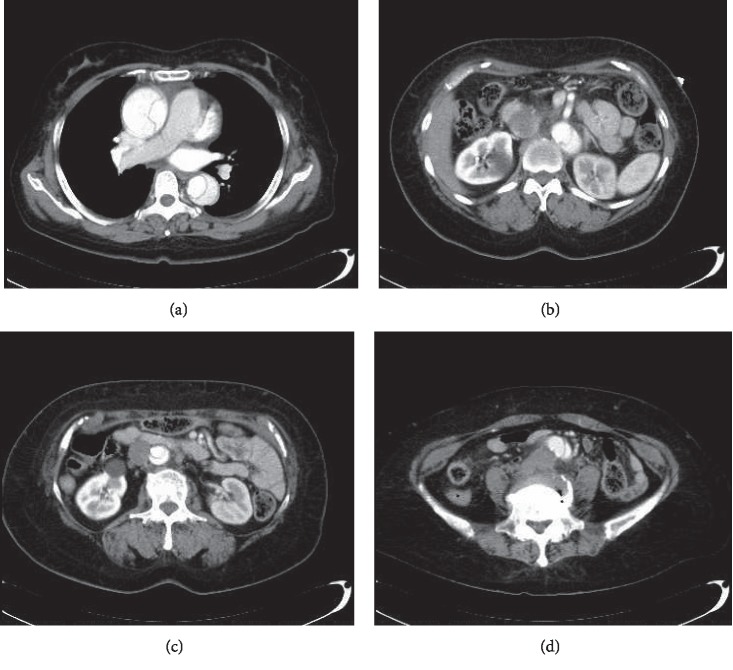
Preoperative enhanced computed tomography (CT) image shows (a) acute type A aortic dissection, (b) superior mesenteric artery perfusion from the true lumen, (c) left kidney not well enhanced, and (d) dissected lumen extending to the terminal aorta.

A 61-year-old woman with a history of untreated hypertension presented to a local physician with sudden back pain. She was diagnosed with ATAAD using enhanced computed tomography (CT), which revealed the presence of a wide-spread patent false lumen from the sinus of Valsalva to the terminal abdominal aorta and poor enhancement of the left kidney (Figure 1). Transthoracic echocardiography revealed moderate aortic valve regurgitation (AR). She was transferred to our hospital. We performed total arch replacement and resuspension of the aortic valve commissures under hypothermia (lowest rectal temperature was 25.7°C) and the lower body circulatory arrest using cardiopulmonary bypass and selective cerebral perfusion. We transfused 6 units of red blood cells (RBC), 26 units of fresh frozen plasma (FFP), and 20 units of platelet concentrates (PC) during the surgery.

**Figure 2:**
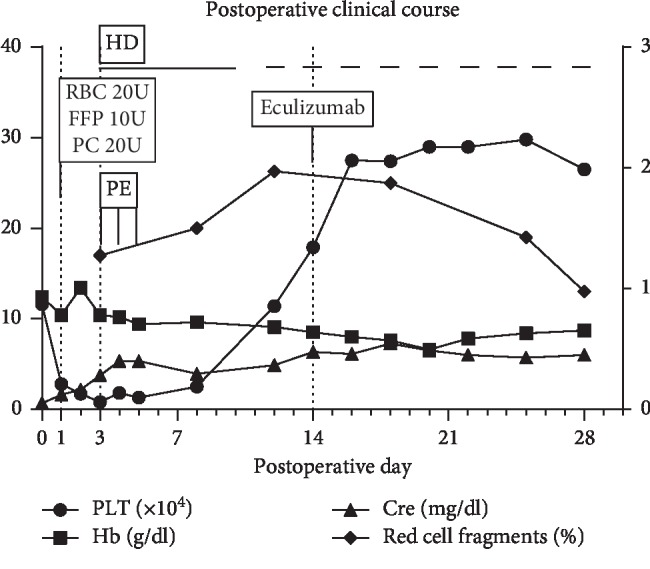
Graph shows the postoperative clinical course. Hb: haemoglobin, PLT: platelet count, Cre: creatinine, RBC: red blood cell, FFP: fresh frozen plasma, PC: platelet concentrates, HD: hemodialysis, and PE: plasma exchange.

On postoperative day 1, the platelet count decreased from 116 × 10^3^/*µ*l to 28 × 10^3^/*µ*l, and we transfused 20 units of PC. On postoperative day 2, her platelet count did not increase. On postoperative day 3, her platelet count further dropped to 8 × 10^3^/*µ*l, and her renal function worsened. On the same day, we confirmed schistocytes in her blood smear. Immediate consultations with the hematology team yielded a diagnosis of thrombotic microangiopathy (TMA), and we initiated plasma exchange (PE) and hemodialysis (HD). Therefore, we investigated for HUS and TTP as the major causes of TMA. Although PE increased the platelet count, renal function did not improve, and the schistocytes continued to increase. The tests for HUS and TTP were negative, and both these conditions were excluded. Furthermore, her medical history, physical examination, and laboratory data were not suggestive of other TMA-related diseases (Table 1). We diagnosed her with aHUS on postoperative day 14 and immediately started eculizumab treatment, following which, hemolytic anemia and thrombocytopenia improved without the need for further eculizumab or transfusion support. However, renal function did not recover, and maintenance hemodialysis was needed.  Figure 2 shows the postoperative clinical course. Subsequently, the patient was transferred to another hospital for rehabilitation. One year postoperatively, she died of multiorgan failure.

## 3. Discussion and Conclusion

Patients presenting with triad of TMA, such as microangiopathic hemolytic anemia, thrombocytopenia, and AKI, and tests positive for STEC infection are diagnosed with HUS [3]. Conversely, patients presenting with TMA and <10% of the normal activity of a disintegrin-like and metalloproteinase with thrombospondin type 1 motifs 13 (ADAMTS-13) or positive for anti-ADAMTS-13 antibodies are diagnosed with TTP. Further, patients with TMA tests negative for STEC infection, TTP, and other known causes of TMA, such as calcineurin inhibitors, sirolimus, anti-VEGF agents, transplantation, cobalamin deficiency, and autoimmune diseases, are clinically diagnosed with aHUS [1]. aHUS is caused by uncontrolled activation of the complement system in the alternative pathway, causing microvascular thrombosis and vascular endothelial injury, and subsequently, microangiopathic hemolytic anemia, thrombocytopenia, and AKI. Gene mutations in complement system proteins such as *CFH*, *C3*, *CFI*, *MCP*, *CFB*, and *THBD* are documented in aHUS patients [4]. aHUS is a rare disease with an estimated incidence of 0.2–2 cases per million [5]. Several definitions for TMA and aHUS have been published. In brief, the diagnosis of aHUS is made based on the triad of TMA and negative results for STEC-HUS, TTP, and other TMA-related diseases, such as autoimmune diseases.

Published reports show poor prognosis, with 33%–40% of aHUS patients dying or progressing to end-stage renal disease (ESRD) during the acute phase and up to 65% of cases progressing to ESRD or dying within 1 year. Traditionally, the treatment for aHUS has been PE. However, recently, a study showed successful treatment of aHUS with eculizumab, a humanised monoclonal antibody that binds to C5 and inhibits the generation of proinflammatory C5a and C5b-9 in the alternative pathway. Eculizumab has been established as the first-line treatment for aHUS [6].

In this case, multiple factors, such as the disease itself and surgical stress, including hypothermia, cardiopulmonary bypass and lower body circulatory arrest, could have triggered the uncontrolled complement activation. Despite immediate treatment with PE following the manifestation of the TMA triad, thrombocytopenia improved, but the progression of schistocytes was not resolved. Further, eculizumab treatment 10 days after PE initiation without additional transfusion suppressed the progression of hemolytic anemia; however, her renal function did not recover enough. An investigation into gene mutations of aHUS is needed for the definitive diagnosis of aHUS when the patient has been clinically diagnosed with aHUS; however, specific mutations in the complement genes are not determined in 40% of patients clinically diagnosed with aHUS [5]. In this case, no significant gene mutations in *CFH*, *C3*, *CFI*, *MCP*, *CFB*, and *THBD* associated with aHUS were detected (Table 2).

Some cases of aHUS have been reported following transplantation surgery, including heart transplantation [7]. Conversely, only two cases of aHUS developing after nontransplantation cardiac surgery have been reported [8,9]. Although Saltzman et al. reported five cases of TTP following open heart surgery, ADAMTS-13 levels in four of those five cases were >10%, and anti-ADAMTS-13 antibody levels were within the normal level [10]. According to the current diagnostic criteria, these four cases might have developed aHUS.

This is the first case report of aHUS following ATAAD. aHUS must be suspected if the patient manifests the triad of TMA during treatment of acute aortic dissection, and the relevant diagnostic workup must be performed.

## Figures and Tables

**Table 1:** Diagnostic tests for TMA-related disease.

Parameters	Results	Normal range
Shiga-toxin-producing *Escherichia coli* infection		
Stool culture	Negative	Negative
O-157 LPS Ab	Not detected	Not detected

Diagnostic tests for TTP		
ADAMTS-13 activity (%)	69.1	70–120
Anti-ADAMTS-13 antibody (BU/mL)	<0.5	<0.5

Diagnostic tests for secondary TMA		
C3 (mg/dL)	96	65–135
C4 (mg/dL)	11	13–35
CH50 (IU/ml)	42.2	30–46
Direct Coombs test	Negative	Negative
Indirect Coombs test	Negative	Negative
Anti-nuclear antibody (titer)	<1 : 20	<1 : 40
PR3-ANCA (IU/ml)	Not detected	<3.5
MPO-ANCA (IU/ml)	Not detected	<3.5
Lupus anticoagulant	Negative	Negative
CL*β*2GPI (IU/ml)	Not detected	<10

O-157 LPS Ab: O-157 lipopolysaccharide antibody, TTP: thrombocytopenic purpura, ADAMTS-13: a disintegrin-like and metalloproteinase with thrombospondin type 1 motifs *13*, PR-3 ANCA: proteinase 3 anti-neutrophil cytoplasmic antibody, MPO-ANCA: myeloperoxidase anti-neutrophil cytoplasmic antibody CL*β*2GPI: anti-cardiolipin *β*2-glycoprotein I complex antibody.

**Table 2:** Analysis of the responsible gene for aHUS.

Responsible gene	Mutation	Comment
CFH	+	Polymorphism
C3	+	Polymorphism
CFI	−	—
MCP	−	—
CFB	+	Variation seen within normal 2% in Japanese
THBD	+	Polymorphism
